# Integration of Untargeted Metabolomics with Transcriptomics Provides Insights into Beauvericin Biosynthesis in *Cordyceps chanhua* under H_2_O_2_-Induced Oxidative Stress

**DOI:** 10.3390/jof8050484

**Published:** 2022-05-06

**Authors:** Cheng Zhao, Haifen Bu, Jiahua Zhu, Yulong Wang, Kerry M. Oliver, Fenglin Hu, Bo Huang, Zengzhi Li, Fan Peng

**Affiliations:** 1 Engineering Research Center of Fungal Biotechnology, Ministry of Education, Anhui Provincial Key Laboratory for Microbial Control, Anhui Agricultural University, Hefei 230036, China; zhaocheg@stu.ahau.edu.cn (C.Z.); haifenb96@163.com (H.B.); zhudiaohua@gmail.com (J.Z.); wyl2019@ahau.edu.cn (Y.W.); hufenglin@ahau.edu.cn (F.H.); bhuang@ahau.eud.cn (B.H.); zzli@ahau.edu.cn (Z.L.); 2 Department of Entomology, University of Georgia, Athens, GA 30602, USA; kmoliver@uga.edu

**Keywords:** *Cordyceps chanhua*, beauvericin, oxidative stress, metabolome, transcriptome

## Abstract

*Cordyceps chanhua* is an important cordycipitoid mushroom widely used in Asia and beyond. Beauvericin (BEA), one of the bioactive compounds of *C. chanhua*, has attracted much attention because of its medicinal value and food safety risk. In order to clear up the relationship between oxidative stress and BEA synthesis, we investigated the impact of H_2_O_2_-induced oxidative stress on the secondary metabolism of *C. chanhua* using untargeted metabolomics and a transcript profiling approach. Metabolic profiling of *C. chanhua* mycelia found that in total, 73 differential metabolites were identified, including organic acids, phospholipids, and non-ribosomal peptides (NRPs), especially the content of BEA, increasing 13-fold under oxidative stress treatment. Combining transcriptomic and metabolomic analyses, we found that the genes and metabolites associated with the NRP metabolism, especially the BEA biosynthesis, were highly significantly enriched under H_2_O_2_-induced stress, which indicated that the BEA metabolism might be positive in the resistance of *C. chanhua* to oxidative stress. These results not only aid in better understanding of the resistance mechanisms of *C. chanhua* against oxidative stress but also might be helpful for molecular breeding of *C. chanhua* with low BEA content.

## 1. Introduction

The fungus *Cordyceps chanhua* (syn. *Isaria cicadae* and *C. cicadae*), verified as a new species of Cordicipitaceae [1], is a parasite of cicadas, but also used in traditional Chinese medicine, particularly in the treatment of kidney disease [2,3]. Modern studies have shown that *C. chanhua* can modulate immune function [4], function as an antioxidant [4,5], and have anti-inflammatory [6] and anti-tumor [7] properties. This fungus has been mass produced on artificial media for use in food supplements or health additives [8]. Many bioactive substances have been identified from *C. chanhua*, including beauvericin (BEA). BEA is a non-ribosomal peptide (NRPs) first isolated from the fungus *Beauveria bassiana* (Syn. *C. bassiana*) and also found in *C. tenuipes*, *Fusarium*, and *Trichoderma* [9,10]. In addition to insecticidal action, BEA also has anti-bacterial, fungal, virus, anti-tumor, ACAT inhibition, and other activities [11,12]. BEA is also genotoxic (producing DNA fragmentation, chromosomal aberrations, and micronucleus) and induces apoptosis of Hep-G2 and U937 cells by participating in the mitochondrial pathway [13]. BEA (0.1 and 0.5 μM) caused a significant DNA damage in PK15 cells, which was dose- and time-dependent [14]. In addition to its pharmacological activity, there is much interest in BEA because of its possible entry into the food chain as a metabolite of *Fusarium*, a common infection of cereals [15]. While acute exposure to BEA poses little concern for human health, chronic exposure of BEA alone, or in combination with other mycotoxins, represents a potential risk for humans and animals [16]. Therefore, BEA has attracted attention both due to its medicinal value and risks to food safety.

The biosynthetic pathway of BEA is relatively clear. D-hydroxyisovaleric acid (D-HIV) and L-phenylalanine (L-Phe) are the key substrates for the synthesis of BEA, and BEA synthase depends on these two substances for activation [17]. However, mechanisms of BEA synthesis and regulation are less clear [18]. For example, using Phe as a nitrogen source does not improve the yield of BEA, but the addition of the precursors L-isoleucine or D-isoleucine does improve yield [19]. In our previous study, we found that mycotoxins, including BEA derived from *C. chanhua* mycelia, accumulate after several generations of subculture. Subculturing also led to the accumulation of reactive oxygen species (ROS) [20]. The production of mycotoxins may be to eliminate the excessive accumulation of ROS on cells and maintain a certain oxidation level in cells to reduce the harm to fungi, which is known as the “oxidative stress theory of mycotoxin biosynthesis” [21]. In some fungi, the addition of oxidants to the culture medium inhibits the growth of the fungi and promotes the production of mycotoxins [22,23]. In contrast, antioxidants can inhibit the production of mycotoxins [23,24]. The induction of oxidative stress by either ROS or ROS-generating compound applications resulted in extensive alterations of the metabolic profiles of fungi, especially with regards to primary metabolism and antioxidant mechanisms [25]. These studies on oxidative stress have focused mostly on aflatoxins, and only one mentioned significant increases of BEA in *B. bassiana* mycelia when adding the oxidative stress agent H_2_O_2_ in liquid culture [26].

In our previous study, we examined the effects of exogenous oxidative stress agents H_2_O_2_ and menadione on *C. chanhua*. We found that some concentrations of H_2_O_2_ significantly promoted the production of BEA while menadione had no effect. We hypothesized that the specific response of *C. chanhua* to H_2_O_2_-induced oxidative stress may play an important role in regulating BEA biosynthesis. Here, we conducted transcriptional and metabolic studies in order to better understand the relationship between oxidative stress and BEA formation.

## 2. Materials and Methods

### 2.1. Fungal Culture and Sample Preparation

*C. chanhua* strain RCEF5833 was isolated from bamboo cicadas *Platylomia pieli* Kato (Hemiptera: Cicadidae) collected in Jingtingshan, Xuancheng in Anhui province, China, and preserved at the Research Center on Entomogenous Fungi (RCEF). Conidial suspensions for inoculation were prepared using fresh conidia harvested from SDAY (glucose 4%, peptone 1%, yeast extract 1%, and agar 2%) after 12 days incubation in the dark at 25 °C ± 1 °C using 0.05% sterilized Tween-80 solution and diluted to the concentration of 1 × 10^7^ conidia/mL. A total of 1 μL of conidial suspension was inoculated onto the center of a 90 mm Petri dish at 25 °C in the dark. RCEF5833 was cultured in media supplemented with 2 mM H_2_O_2_; media without H_2_O_2_ was used as the control (CK). Three biological replicates were performed for each treatment. Colony diameter was measured at the 12th day, and three plugs with a diameter at 4 mm were picked at 1.0 cm from center of the colony. These plugs were homogenized individually in 0.05% Tween-80, and the conidia were counted to calculate the conidia density (conidia/cm^2^) on the culture surface.

A total of 100 μL of the above conidial suspension were inoculated onto culture covered with cellophane in a Petri dish, spread evenly, and cultured at 25 °C in the dark for 6 days. The mycelia were collected, freeze-dried, and crushed into powder and then kept at 4 °C until extraction. A total of 200 mg lyophilized powder was extracted with 500 μL methanol and filtered through a 0.22 μm polyvinylidene fluoride (PVDF) membrane before HPLC–MS or HPLC analysis.

### 2.2. LC–MS Data Acquisition

The extract was analyzed using Agilent 1100 (Agilent Technologies, Inc., Palo Alto, CA, USA) equipped with a photodiode array detector and a HPLC-QTOF-MS (6210 Time of Flight) system equipped with an electrospray ionization source (ESI). We used the tuning standard (Triton X-100) solution to tune the instrument to calibrate the instrument. The absolute value of ppm in the tuning report was less than 3, indicating that the instrument was successfully calibrated [27]. The samples were analyzed separately in both positive and negative ionization modes. The mobile phases were distilled water (A, containing 0.1% formic acid, Agilent, Palo Alto, USA) and acetonitrile (B, Merck, Darmstadt, Germany, containing 0.1% formic acid,); elution conditions: 0–3 min, 5% B; 3–10 min, 5–50% B; 10–39 min, 50–100% B; 39–49% min, 100% B; injection volume, 5 μL; column temperature, 30 °C; flow rate, 0.3 mL/min; the column was an Agilent poroshell 120EC-C18 column (2.7 μm, 3.0 × 100 mm). The eluate was monitored with a photodiode array detector, and the full-wavelength scan was performed from 200 to 600 nm. The mass spectrometer parameter setting refers to the work of Luo [28] and He et al. [29].

### 2.3. Data Processing and Metabolites Identification

The raw LC–MS data were automatically detected by the Molecular Feature Extraction (MFE) algorithm of MassHunter software (Agilent Technologies, Inc., Palo Alto, CA, USA) for automatic peak detection and chromatographic deconvolution (Deconvolution). Peaks with a signal-to-noise (S/N) ratio <5 would be rejected, and the absolute peak height was greater than 5000, so from this, the peak table of each sample would be derived. The data were uploaded to MetaboAnalyst for processing and statistical analysis [29]. Principal component analysis (PCA) and orthogonal partial least squares discriminant analysis (OPLS-DA) were carried out on SIMCA-P 14.1(Umetrics, Umeå, Sweden). Metabolite identification was performed according to the approach of Luo et al. [28]. The molecular formula was calculated by Masshunter on the basis of accurate mass and isotopic pattern recognition. Compounds were putatively identified by searching the molecular formulas against the in-house database of entomopathogenic fungi and web databases (DNP, METLIN, etc.). The mass fragments of metabolites were compared with the compound fragments in METLIN and MASSBANK and verified by the elution order (polarity) and structural properties. Ambiguous metabolites were identified by comparison with available authentic compounds and/or by referring to the published literature about fungi, especially entomopathogenic fungi.

### 2.4. Total RNA Extraction, Library Establishment, and Transcriptome Sequencing

Total RNA was isolated from mycelia of *C. chanhua* cultured on the sixth day. Total RNA was extracted from two groups of samples using Trizol reagent, and the integrity of RNA was evaluated by ND-1000 nucleic acid analyzer (Bio-rad Laboratories, Inc., Berkeley, CA, USA) for subsequent experiments. Total RNA was treated by mRNA enrichment or rRNA removal method. RNA was fragmented by interrupt buffer, and random N6 primers were used for reverse transcription, and then double-stranded DNA was synthesized from two strands of cDNA. The end of the synthesized double-stranded DNA was flattened and phosphorylated at the 5′ end. The 3′ end formed a sticky end with an “A” protruding, and then a bubbly linker with a protruding “T” at the 3′ end was connected. The ligation product was amplified by PCR with specific primers. The PCR product was thermally denatured into single-stranded form, and then the single-stranded DNA was circularized with a bridge primer to obtain a single-stranded circular DNA library, which was then sequenced on the computer.

### 2.5. Transcriptome Assembly and Bioinformatics Analysis

We first filtered out low-quality reads with contaminated linkers and high percentage of unknown bases, and clean reads were obtained and saved in the FASTQ format. These reads were assembled through Trinity, and then Tgicl (a software, v2.1) was used to cluster the transcripts to de novo assembly to obtain Unigene. Then Unigene was functionally annotated and SSR (simple sequence repeat)-tested, and then the expression level of each sample was calculated on the basis of All-Unigene. For multiple samples, differentially expressed genes between different samples were tested according to requirements, and in-depth cluster analysis and functional enrichment analysis were performed on differentially expressed genes. By using Bowtie2 (a software, v2.3.4.1), clean reads were aligned to the genome sequence, and then RSEM (a software package for RNA SEQ reads to calculate the expression of genes and transcript subtypes, v1.2.8) was used to calculate the gene expression level of each sample. RSEM was used for RNA-seq reads to calculate the expression level of genes and transcript subtypes. The gene expression level was determined by calculating the number of fragments in each sample and standardized to FPKM (fragments per kilobase of exon model per million mapped fragments).

### 2.6. GO Function Classification and KEGG Pathway Enrichment Analysis of DEGs

Differentially expressed genes (DEGs) were detected according to the method described by Michael et al.; the DEseq2 method was based on the principle of negative binomial distribution [30]. In order to further analyze the DEG annotations, on the basis of the GO database and the KEGG database, the functional classification of differential genes was carried out, and the phyper function in the R software (v2.3.4) was used for enrichment analysis.

### 2.7. Quantitative Real-Time PCR (qRT-PCR) Validation

The qRT-PCR template cDNA was synthesized from total RNA. Each qRT-PCR reaction system had a total volume of 20 μL, including 2 μL of c DNA, 4 μL of upstream and downstream primers, 7.2 μL of ddH_2_O, and 10 μL mixture of 2 × SYBR Green Master Mix (Vazyme Biotech Co., Ltd, Nanjing, China). All qRT-PCR reactions were carried out in a CFX96 Real-Time System (Bio-rad Laboratories, Inc., Berkeley, CA, USA), referring to the reaction parameters in the SYBR Green Master Mix (Vazyme Biotech Co., Ltd, Nanjing, China) guide. The actin gene was used as internal reference gene, and the relative gene expression level was calculated by the 2^−△△^CT method [31].

### 2.8. BEA Production Assay

A total of 200 mg of lyophilized mycelia powder was extracted with methanol and filtered through a 0.22 μm polyvinylidene fluoride (PVDF) membrane before HPLC detection. For detection and quantification of BEA, HPLC, LC-20AD system (Shimadzu (China) Co., Ltd., Shanghai, China) was performed using a UV detector set at 215 nm equipped with a C18 reversed-phase column (2.1 × 150 mm, 5 μm, Agilent Technologies, Inc., Palo Alto, CA, USA ). The mobile phase was acetonitrile/water (70:30) for 30 min, at a flow rate of 0.3 mL/min. Injection volume was 10 μL, and column temperature was 30 °C. BEA was determined by UV detection at a wavelength of 215 nm. A standard BEA curve was generated using 30~270 μg/mL BEA standard (Sigma-Aldrich, Burlington, MA, USA). The BEA yield was calculated using the detected peak area according to the standard curve. The BEA concentration of mycelia presented in our study was calculated by normalizing in the equal biomass.

## 3. Results

### 3.1. Effect of H_2_O_2_ on Metabolites of C. chanhua

**Figure 1:**
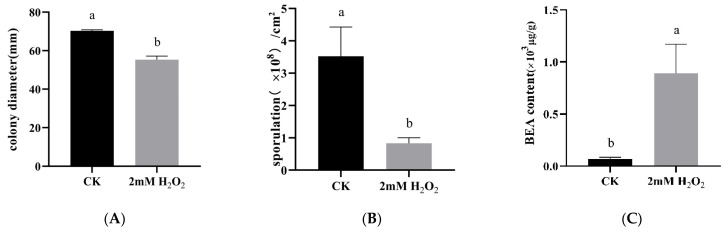
(**A**) Effect of H_2_O_2_ on the growth of *C. chanhua*. (**B**) Effect of H_2_O_2_ on the sporulation of *C. chanhua*. (**C**) Effect of H_2_O_2_ on the BEA content of *C. chanhua.* CK, control, without H_2_O_2._ Different Lower letters indicate significant difference (*p* < 0.05).

**Figure 2:**
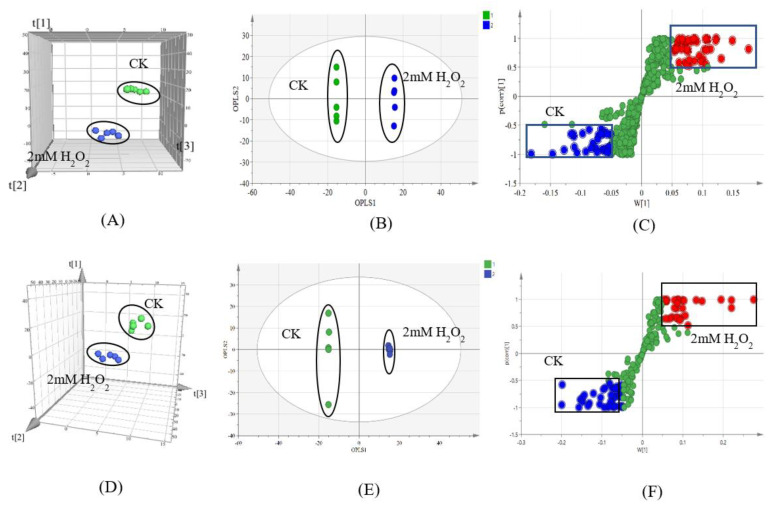
(**A**) PCA score plots according to HPLC−MS in positive ionization mode (PC_1_ = 47.6%, PC_2_ = 17.1%, and PC_3_ = 13.0%). (**B**) OPLS−DA score plots in positive ionization mode (R^2^x = 0.601, R^2^y = 1, Q^2^ = 0.957). (**C**) S-plot based on OPLS−DA model in positive ionization mode. (**D**) PCA score plots based on HPLC–MS in negative ionization mode (PC_1_ = 37.3%, PC_2_ = 14.5%, and PC_3_ = 10.6%). (**E**) OPLS−DA score plots in negative ionization mode (R^2^x = 0.726, R^2^y = 1, Q^2^ = 0.928). (**F**) S−plot based on OPLS−DA model in negative ionization mode. CK, control, without H_2_O_2._

**Figure 3:**
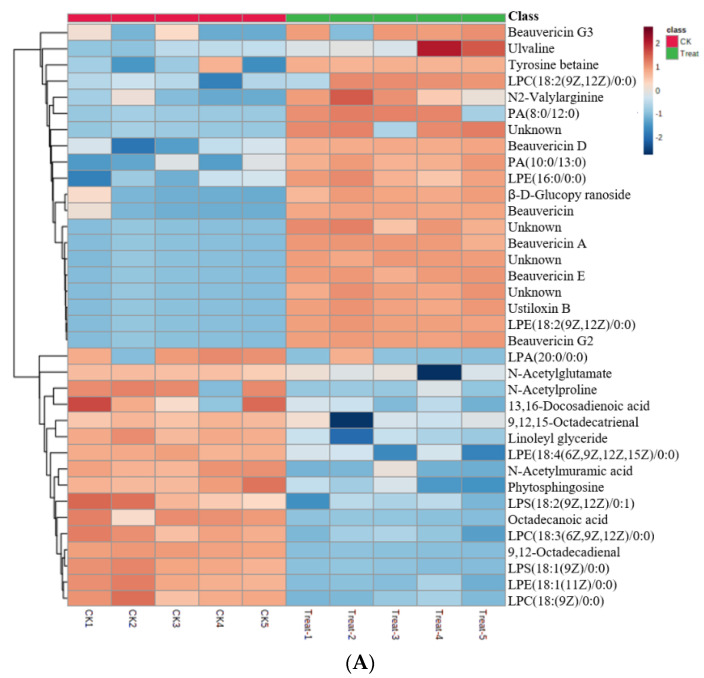

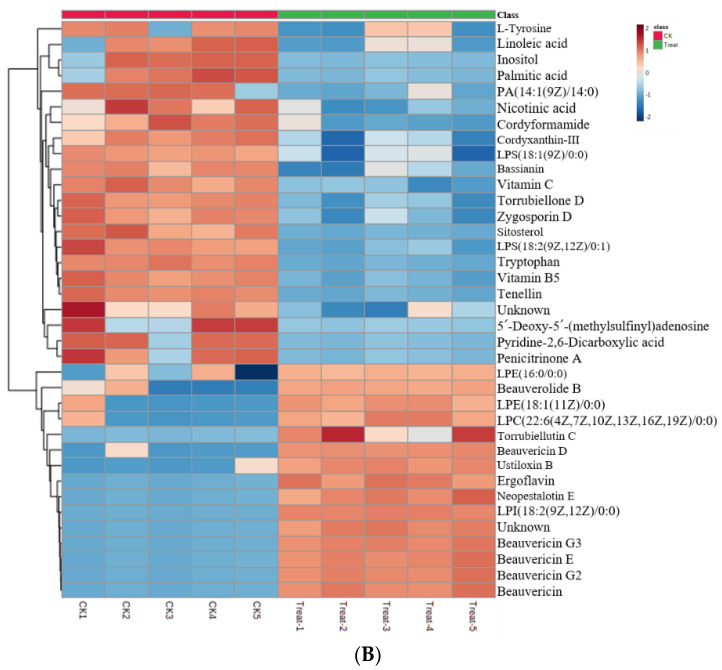
(**A**) Heat map of differential metabolites in positive ionization mode under oxidative stress induced by H_2_O_2_. (**B**) Heat map of differential metabolites in negative ionization mode under oxidative stress induced by H_2_O_2_. CK, control, without H_2_O_2_; Treat, with H_2_O_2_, and 1−5 represent five biological repetitions.

Compared to controls, oxidative stress induced by 2 mM H_2_O_2_ diminished the growth of *C. chanhua* (Figure 1A, *p* < 0.05), reducing the sporulation by more than twofold (Figure 1B, *p* < 0.05). The metabolomic profiles of *C. chanhua* grown in H_2_O_2_ treatment were also different from the controls, which implies condition-dependent nutrient consumption and metabolite release. In Figure 2A,D, the clearly separated PCA score plots are shown. A supervised pattern recognition method OPLS-DA was used to discriminate differential metabolites of the treatment versus control (Figure 2B,E). Different metabolites between the two groups were identified by S-plot at |w (1)| and |p (corr)|, which were ≥0.05 and 0.5, respectively, and at a *p*-value < 0.05 according to OPLS-DA models (Figure 2C,F). A visual hierarchical clustering analysis (HCA, Figure 3) was performed to simultaneously identify the metabolic discrepancies under different conditions. As Figure 2 shows, HCA included 37 metabolites in positive ionization mode (Figure 3A) and 36 metabolites in negative ionization mode (Figure 3B) that were significantly different (*p* < 0.01) at one-way ANOVA, mainly including organic acids, phospholipids, and non-ribosomal peptides (NPRs). The changes in the level of these metabolites indicated that *C. chanhua* adapts to oxidative stress through self-regulation. The organic acids and energy supply substances in the treatment group were significantly lower than those in the control. Phospholipids are the basic unit for constructing biological membranes. Compared with the control, PA and LPA, LPC, LPE, and LPS, the four lysophospholipids in the treatment, all increased or decreased at certain levels. In this study, a total of seven NRPs in *C. chanhua* under oxidative stress were detected as being higher than those in the control. In addition to beauvericin (BEA), there were beauvericin A, beauvericin D, beauvericin E, beauvericin G2, beauvericin G3, and beauverolide B. BEA content was further characterized by HPLC, showing a 13-fold increase in production (889.6 μg/g) under oxidative stress, compared to the control (69.7 μg/g) (Figure 1C).

### 3.2. Transcriptomics Analysis of C. chanhua under Oxidative Stress

A transcriptomics analysis was conducted using Illumina RNA-Seq to compare gene expression profiles in H_2_O_2_-treated cultures versus the control. Each sample produced an average of 6.39 Gb of total clean bases, with 47.62 M raw reads (Supplementary Table S1). The total clean reads after screening and filtering averaged 42.60 M, and the percentage of bases with a mass value greater than 30 in the filtered reads of each sample accounted for more than 85% of the total bases. A total of 24,240 Unigenes with a total length of 52.24 M and a GC content ratio of 56.05% were obtained.

### 3.3. Differentially Expressed Genes (DGEs) in C. chanhua

**Figure 4:**
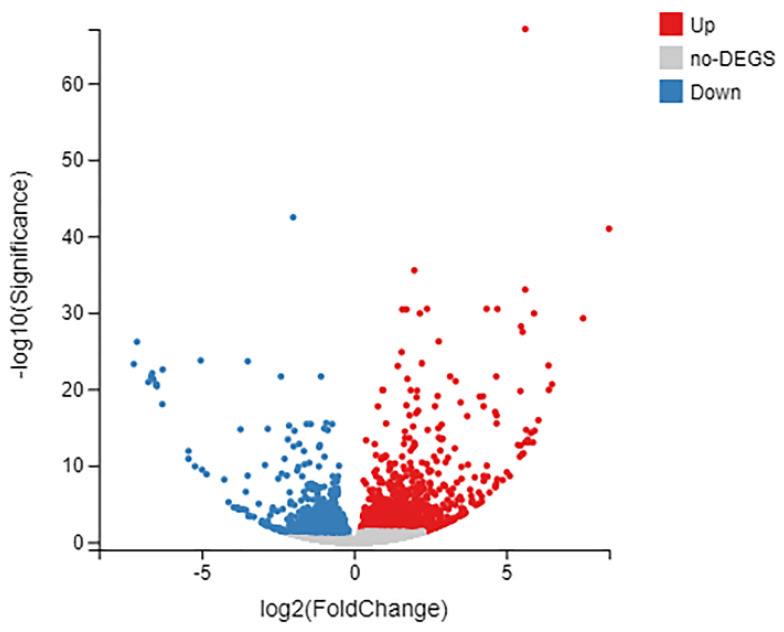
Volcano plots of total gene expression profiles between the two compared groups. Each dot represents the mean expression of individual genes obtained from the transcriptome. Genes above the cut-off lines were considered as DEGs, which were defined as those with fold change ≥ 2 and FDR < 0.05 and illustrated in red (increased expression) or blue (decreased expression); non−DEGs are illustrated in gray.

On the basis of the TPM values and using the adjusted *p*-value < 0.05 and |log_2_ (Fold Change)| ≥ 1 as thresholds, the DEGs of the treatment and control groups were identified [30]. A total of 1983 DEGs were identified and are shown in a volcano plot (Figure 4, Supplementary Table S2). Compared to the control, 1210 genes were upregulated and 773 genes were downregulated in the H_2_O_2_ treatment. As shown in the volcano plot (Figure 4), some of the DEGs displayed significant differences.

### 3.4. GO Analysis and KEGG Pathway Enrichment of DEGs

**Figure 5:**
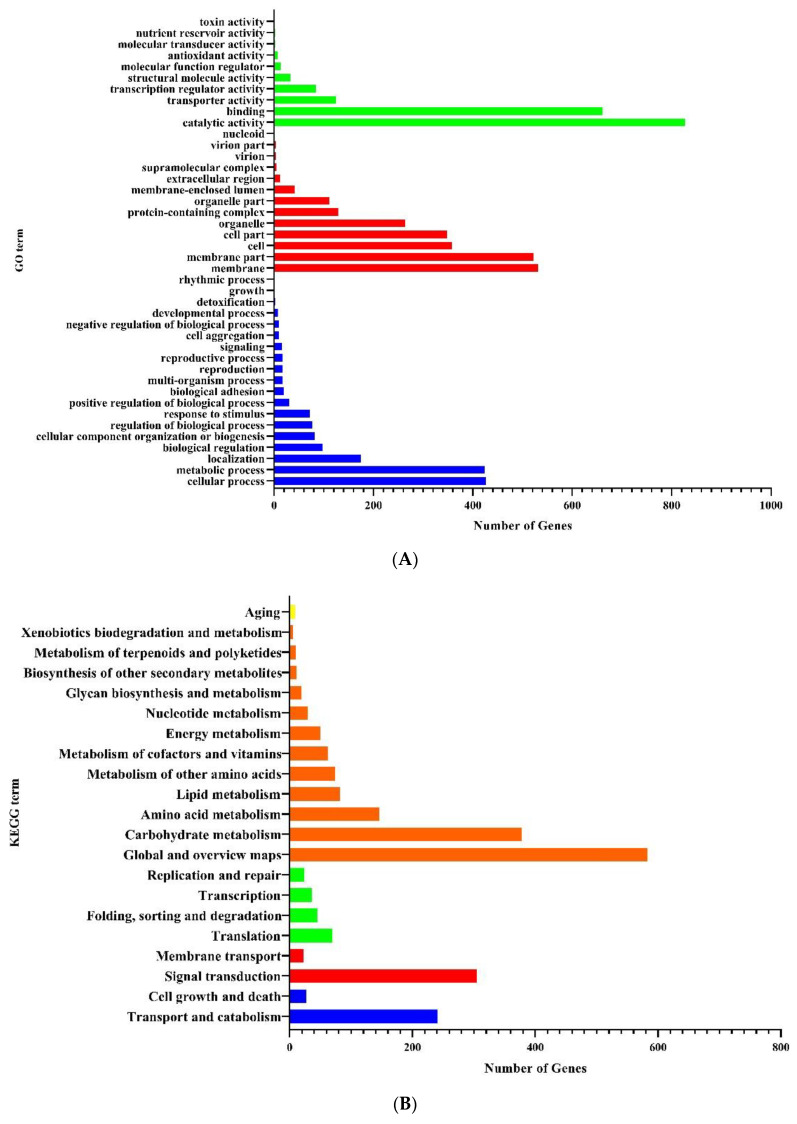
Functional classification of differentially expressed genes. (**A**) DEGs were classified according to the GO database (*X*-axis represents the number of DEGs, *Y*-axis represents GO term; green, molecular function; red, cellular component; blue, biological process; GO, Gene Ontology). (**B**) DEGs are classified according to the KEGG database (*X*-axis represents the number of DEGs and *Y*-axis represents KEGG terms; yellow, organic systems; orange, metabolism; green, genetic information processing; red, environmental information processing; blue, cellular processes; KEGG, Kyoto Encyclopedia of Genes and Genomes).

GO classification and functional enrichment were performed for gene annotation. There were 645, 816, and 1174 DEGs assigned to biological processes, cellular components, and molecular functions, respectively (Figure 5A). The significantly enriched categories were cell processes, metabolic processes, cells, cell parts, membranes, membrane parts, organelles, binding, and catalytic activity in the comparison between the H_2_O_2_ treatment and the control. Most of the DEGs were classified into GO terms including biological processes “cell cortex”, “integral component of membrane”, “transmembrane transport”, “oxidoreductase activity”, “phospholipid biosynthesis process”, “carbohydrate metabolism process”, “carbohydrate catabolic process”, “response to oxidative stress”, “peroxidase activity”, “aromatic amino acid family biosynthetic process”, and “mycotoxin biosynthesis process”, of which 506 genes were located in the “integral component of membrane”. The above results suggest that the damage to *C. chanhua* induced by H_2_O_2_ could be reduced by regulating the biological processes such as cell membrane composition, transmembrane transport process, enzyme activity, and metabolite synthesis or decomposition.

To further verify the GO ontologies results, GO functional enrichment of DEGs was performed in the KEGG database. All the genes were mapped to categorize the biological functions of the DEGs on the basis of the KEGG database. Twenty-one pathways showed DEGs enrichment (1082 genes), and the cluster “Global and overview maps” (583 genes, 53.88%) was the largest group. Clusters for “Carbohydrate metabolism” (378 genes, 34.93%), “Signal transduction” (305 genes, 28.19%), “Transportation and catabolism” (241 genes, 22.27%), and “Amino acid metabolism” (146 genes, 13.49%) were followed (Figure 5B). Moreover, a *p*-value of 15 pathways was less than 0.05 (Table 1). Pathways “MAPK signaling pathway”, “Amino sugar and nucleotide sugar metabolism,” “Sulfur metabolism”, “Sphingolipid metabolism”, “Cyanoamino acid metabolism”, “Arachidonic acid metabolism”, and “Biosynthesis of antibiotics” showed an even greater significant enrichment (*p* < 0.01).

### 3.5. BEA Synthesis Pathway

**Figure 6:**
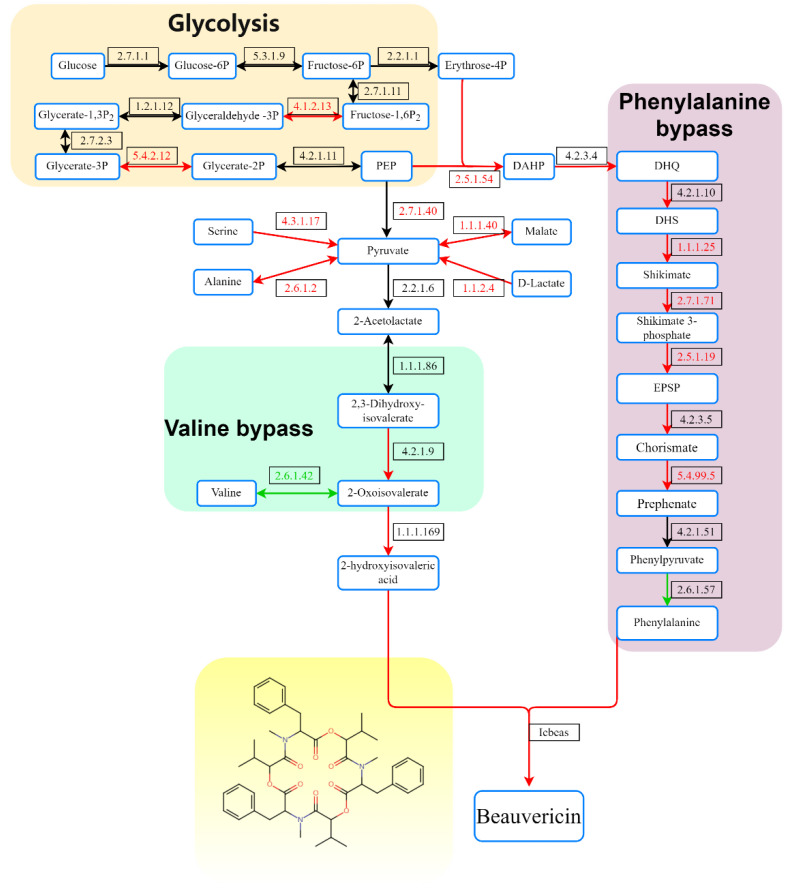
Metabolic and biosynthetic pathways of BEA in *C. chanhua*. PEP, phosphoenolpyruvate; DAHP, 3-deoxy-a-arabinoheptulonic acid-7-phosphate; DHQ, dihydroquinoline; DHS, dehydroshikimic acid; EPSP, 5-enolpyruvylshikimic acid. Shaded to indicate upregulation of the gene or pathway (red, upregulated; green, downregulated; black, unchanged).

In our metabolic analyses, we found that NRP content, including BEA, was higher under oxidative stress than in the controls. We further studied the direct responses of the *C. chanhua* transcriptome to oxidative stress, as well as the effects during BEA production. In the glycolysis pathway (Figure 6, Table 2), the genes of three enzymes were significantly upregulated. Among them, CL2518.Contig2 encoded fructose bisphosphate aldolase (EC 4.1.2.13), CL863.Contig2 encoded phosphoglycerate mutase (EC 5.4.2.12), and Unigene4061 encoded pyruvate kinase (EC 2.7.1.40). These upregulations of DEGs in the glycolysis pathway indicated that H_2_O_2_ promoted the metabolism of substance and energy in *C. chanhua* in which phosphoenolpyruvate (PEP) was the precursor of phenylalanine synthesis and pyruvate was the precursor of valine synthesis.

Pyruvate was the starting compound for valine synthesis, and pyruvate kinase (Unigene4061) was upregulated (treat: 447.2, control: 271.9, 0.76-fold) in the treatment group. In addition, there are two amino acids that can be used as precursors of pyruvate. Serine was dehydrated and deaminated to generate pyruvate; the gene Unigene1709 (treat: 61.1, control 20.4, 1.58-fold) labeled L-serine/L-threonine ammonialyase (EC 4.3.1.17) was significantly upregulated. Alanine generates pyruvate through deamination, the gene CL1908. Contig3 (treat: 24.6, control: 18.1, 0.49-fold) labeled as alanine aminotransferase (EC 2.6.1.2) was upregulated. The upregulation of these two DEGs indicated that H_2_O_2_ promoted the conversion of serine and alanine into pyruvate. In addition, malic acid and D-lactic acid can also be used as precursors of pyruvate. Malic acid undergoes decarboxylation to generate pyruvate under the action of malate dehydrogenase (EC 1.1.1.40). In this study, CL1818.Contig5 was labeled as EC 1.1.1.40, and its transcription level was upregulated by 1.47; CL800.Contig2 was labeled as lactate dehydrogenase (EC 1.1.2.4), which can catalyze the dehydrogenation of D-lactate to pyruvate, and its transcription level was upregulated by 0.93. In the suborder reaction of valine production, 2,3-dihydroxy-3-methylbutyric acid was catalyzed by dihydroxy acid dehydratase to produce 2-keto-3-methylbutyric acid. Two enzymes were annotated as 3-deoxy-7-phosphoheptulonate synthase (EC 2.5.1.54), namely, unigene5252 (treat: 7.6, control: 4.0, 0.99-fold) and CL146.contig5 (treat: 2.3, control: 1.1, 1.12-fold). Among them, 2-keto-3-methylbutyric acid was a compound directly involved in the synthesis of BEA in the synthesis pathway of valine; CL4308.Contig1 was annotated as branched-chain amino acid transaminase (EC 2.6.1.42), as the last step in the synthesis of valine, which catalyzed 2-keto-3-methylbutyrate acid to produce valine. In this study, the transcription level of the gene was significantly decreased (treat: 40.4, control: 54.5, 0.38-fold), indicating that the production of valine from 2-keto-3-methylbutyric acid was significantly inhibited.

PEP and erythrose 4-phosphate (Erythrose-4P) were the starting compounds for the synthesis of aromatic amino acids. Both were catalyzed by 3-deoxy-7-phosphoheptanone synthase (EC 2.5.1.54) to synthesize 3-deoxy-α-arabino-heptulonic acid-7-phosphate (DAHP). CL2696.contig4 and CL2696.contig3 were labeled as EC 2.5.1.54. The transcription levels of the two DEGs in the treatment were 2.0 and 3.7 times higher than in the control. Pentafunctional AROM polypeptide is a five-functional polypeptide that can catalyze five consecutive reactions to produce 5-enolpyruvylshikimic acid in the shikimate pathway [32]. In this study, Unigene 3610 was labeled as Pentafunctional AROM polypeptide (EC 4.2.3.4), and its transcription level increased significantly (treat: 38.2, control: 20.0, 0.99-fold). Since chorismate is the branch point of the aromatic amino acid synthesis pathway, it can be converted into tryptophan precursor compound anthranilate by anthranilate synthase (EC 4.1.3.27), or into phenylalanine precursor compound prephenic acid by chorismate mutase (EC 5.4.99.5). The results showed that there was no significant difference between the genes expressing EC 4.1.3.27, but the transcription level of the gene CL2040.Contig1, which was labeled as EC 5.4.99.5, increased significantly, indicating that chorismate was more converted into prephenic acid.

Ketoisovalerate reductase (Unigene1653; kivr, EC1.1.1.169) is located in the BEA synthesis gene cluster and can catalyze the production of 2-ketoisovalerate to 2-hydroxyisovalerate. The transcriptional level of this gene in the treatment group was 26.8 times higher than that of the control (treat: 42.9, control: 1.6, 3.97-fold). NRPSs are the last and most important enzymes in the BEA synthesis pathway. They consist of two modules, and their structure conforms to the characteristics of C1A1T1-C2A2(M)T2aT2b-C3. The A1 and A2 domains recognize, activate, and connect phenylalanine and 2-hydroxyisovaleric acid, respectively, and after partial reaction, dipeptidol monomers are formed under the action of the C2 domain, and BEA is formed by three cycles of cyclization [33]. A total of three genes—Unigene6975, Unigene3578, and Unigene2977—were labeled as NRPSs in the NR database, and Unigene6975 and Unigene2977 were labeled as partial mRNAs of NRPS genes in the NT database. The Unigene3578 gene was not annotated in the NT database. The length of the gene was only 610 bp, which was much shorter than the length of the NRPSs gene, indicating that it should also be the partial mRNA of NRPSs gene. The expression levels of the three genes in the treatment group were significantly higher than those in the control, and they were all 10 times higher than the control.

### 3.6. qRT-PCR Validation

**Figure 7:**
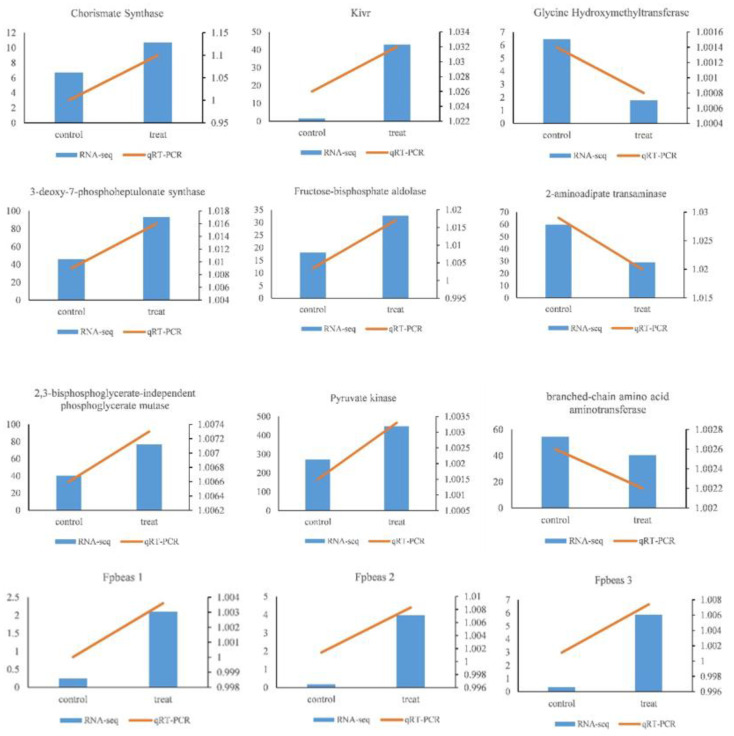
Quantitative real-time PCR. The qRT-PCR was carried out in biological triplicate. The blue bar indicates the relative expression of the genes in RNA-Seq data, and the yellow broken line indicates the relative expression of the genes in qRT-PCR data. The left ordinate represents the gene expression in RNA-Seq, and the right ordinate represents the gene expression in qRT-PCR.

To validate the RNA-seq data, 12 genes across different pathways were selected to perform qRT-PCR. As shown in Figure 7, the transcriptional expression profiles of these selected unigenes were consistent with the transcriptome sequencing results, indicating that the DEGs and pathways identified by RNA-Seq were reliable for understanding the response of *C. chanhua* to oxidative stress induced by H_2_O_2_.

## 4. Discussion

The biosynthesis of BEA depends on several environmental factors including the substrate, pH, temperature, water activity, and their interactions. For example, the yield of BEA in *F. oxysporum* was higher when the carbon source was glucose, the organic nitrogen source was peptone, and the inorganic nitrogen source was NaNO_3_ [34,35]. The precursors of BEA are valine and phenylalanine [17], but in a medium with valine and phenylalanine as nitrogen sources, the production of BEA did not increase [36]. However, adding H_2_O_2_ or methionine to *B. bassiana* cultures did increase BEA production [37]. In this study, the production of BEA in *C. chanhua* increased significantly when 2 mM H_2_O_2_ was added to solid medium (SDAY), which was approximately 13 times higher than the control at the sixth day. It indicated that oxidative stress induced by H_2_O_2_ significantly affected the growth and metabolism of *C. chanhua* and thus affected the metabolism of BEA. Our previous study showed that the content of BEA in *C. chanhua* increased significantly in the later stage of subculture. It was speculated that this might be a stress response to ROS accumulation.

The effect of H_2_O_2_ on the metabolism of *C. chanhua* was further analyzed through metabolomics. As shown in the PCA and OPLS-DA analyses (Figure 2), there were significant differences between the treatment and the control. The small molecules produced by fungi play essential roles in fungus–environment interactions. As a natural antioxidant, vitamin C can directly remove excessive ROS in cells and avoid damaging bio-macromolecules such as fat, protein, and nucleic acid [38,39]. Niacin (vitamin B3) is considered an important antioxidant that affects multiple pathways related to cell survival and death [40]. During oxidative stress, niacin protects cells by blocking inflammatory cell activation, early apoptotic phosphatidylserine exposure, and late nuclear DNA degradation [41]. Pantothenic acid can protect cell membranes from lipid peroxidation damage, as well as protect the plasma membrane from the damage by oxygen free radicals due to increasing cellular level of CoA [42,43]. In the study, KEGG enrichment showed that under the oxidative stress, 28 and 22 genes were enriched in “tryptophan metabolism” and “tyrosine metabolism” pathways, respectively. From the results of the free amino acid assays, the contents of tryptophan and tyrosine did decrease significantly (Supplementary S1). Tryptophan can react with ROS as a reducing agent [44]. Tyrosine contains a phenolic hydroxyl group on the benzene ring, so it has a certain degree of reduction. In vitro experiments have shown that tyrosine has the ability to scavenge H_2_O_2_ [45]. The decrease in the content of these antioxidants in *C. chanhua* indicated that mycelia reduced the oxidative stress by consuming antioxidants. Oxidative stress may be partially countered by invoking changes in cell signaling by free radical scavengers and antioxidants.

Glucose is the starting point of the glycolysis pathway, and its metabolites at all levels are the precursors of many compounds in cells. Glucose, palmitic acid, and linolenic acid can produce acetyl-CoA through different ways, as well as produce a great of ATP through the tricarboxylic acid cycle. It was reported that adding H_2_O_2_ inhibited the absorption rate of glucose and ammonia, also inhibited the activity of glyceraldehyde-3-phosphate dehydrogenase (GAPDH), and decreased the levels of intracellular ATP and NADPH in *Aspergillus niger* [46]. In this study, GO enrichment also showed many genes involved in energy metabolism, such as carbohydrate metabolism process (*p* < 0.05, 47 genes), carbohydrate catabolism process (*p* < 0.05, 4 genes), and tricarboxylic acid cycle (*p* > 0.05, 9 genes), were enriched, and KEGG enrichment showed genes in pyruvate metabolism (*p* < 0.05, 30 genes), fatty acid degradation (*p* < 0.05, 22 genes), starch and sucrose metabolism (*p* > 0.05, 37 genes), glycolysis/gluconeogenesis (*p* > 0.05, 22 genes), and tricarboxylic acid cycle (*p* > 0.05, 10 genes), were upregulated in treated cultures versus controls. The enhancement of energy metabolism can be used to repair cell damage [26] and also increase the production of secondary metabolites such as BEA.

As reported, in addition to amino acids and carbohydrate, glutathione, and polyamine metabolism are also key to *A. flavus* oxidative stress responses [25]. In this study, HCA showed significant changes in phospholipids in the H_2_O_2_ treatment group. Glycerophospholipid, a kind of phospholipid, is one of the main components of the cell membrane and is involved in protein recognition and signal transduction [47]. It was found that bacteria show changes in the phospholipid composition in extreme environments [48,49]. When *Escherichia coli* are cultured in medium containing H_2_O_2_, a small amount of Cu^2+^ and Fe^3+^ can activate the protective mechanism from H_2_O_2_-induced oxidative damage by regulating the phospholipid composition of the membrane [50]. In this study, GO enrichment showed the discrimination genes between H_2_O_2_ treatment and the control related to phospholipid metabolism were enriched in some biological pathways such as “membrane components” (*p* < 0.05), “transmembrane transport” (*p* < 0.05), “phospholipid biosynthesis” (*p* < 0.05), “phosphodiesterhydrolase activity” (*p* > 0.05), and “triglyceride lipase activity” (*p* > 0.05). KEGG enrichment showed the genes were enriched in “glycerin phospholipid metabolism” (*p* > 0.05, 18 genes), “phosphoinositol metabolism” (*p* > 0.05, 13 genes), and “fatty acid metabolism” (*p* > 0.05, 13 genes). H_2_O_2_ does not diffuse freely across cell membranes [51]. Cells respond and adapt to H_2_O_2_ by the regulation of the plasma membrane permeability to H_2_O_2_ [52], and changes of cell membrane components in *C. chanhua* altered the permeability of the cell membrane [53], wherein the polarity and stability of cell membrane was enhanced accordingly, preventing H_2_O_2_ or ROS from entering cells and protecting cells from oxidative stress.

H_2_O_2_ has strong oxidizing properties and generates free radicals in cells. In this study, H_2_O_2_ had a large effect on the metabolism and transcription of *C. cicadae*. The results of GO enrichment showed that most genes were enriched in “peroxidase activity” (*p* < 0.05, 4 genes), “response to oxidative stress” (*p* < 0.05, 6 genes), “DNA repair” (*p* > 0.05, 13 genes), “redox homeostasis” (*p* > 0.05, 7 genes), and other biological process terms. KEGG enrichment showed that “pyruvate metabolism” (*p* < 0.05, 30 genes) and “glutathione metabolism” (*p* < 0.05, 15 genes) were significantly enriched. The DNA of the cells undergoing oxidative damage may be broken, mutated, and changed in terms of thermal stability, which seriously affects the normal transcription and translation process [54]. A total of 13 genes were enriched in the biological process of “DNA repair”, indicating that H_2_O_2_ caused damage to the fungal DNA. Many organisms initiate a signal cascade, leading to upregulation of antioxidant gene transcription in response to oxidative stress [53]. Antioxidant enzymes are considered to be the first line of defense in response to oxidative stress in filamentous fungi [26]. Glutathione is a small molecule antioxidant that can scavenge free radicals and protect cell macromolecules from oxidative damage. In *C. chanhua*, the enrichment results showed that antioxidant enzyme activity and the biological process of glutathione metabolism were significantly enriched. The high-aflatoxin-producing *A. flavus* isolate showed extensive stimulation of antioxidant mechanisms and pathways including glutathione metabolism under oxidative stress [25]. In addition, the pyruvate metabolism pathway was significantly enriched in this study. It was reported that oxidative and osmotic/salt stress can promote pyruvate accumulation, which can remove reactive oxygen species induced by stress [55]. H_2_O_2_-induced stress may trigger defense mechanisms related to the respiratory chain [46]. Pyruvate were effective in reducing oxidative stress by activating the alternative respiratory pathway as well as antioxidant activity in plants [56]. Pyruvate is located at a key intersection in the network of metabolic pathways [57]. Upregulated DEGs in pyruvate metabolism have a great effect on the network of metabolic pathways, and then affect the synthesis of BEA.

When *A. flavus* was treated with tert butyl hydrogen peroxide or gallic acid, aflatoxin increased significantly [58]. The same stress treatment to *A. parasitic* also induced the production of aflatoxin [24]. In our study, GO enrichment showed that “the biosynthesis process of mycotoxins” (*p* < 0.05, 8 genes) was significantly enriched. In KEGG enrichment, “amino acid biosynthesis” (*p* < 0.05, 49 genes) and “aromatic amino acid biosynthesis” (*p* > 0.05, 9 genes) were enriched in many genes. Our amino acid assay results also confirmed that the content of some amino acids such as cysteine, phenylalanine, glutamate, isoleucine, histidine, threonine, valine, and tryptophan were decreased or increased significantly under oxidative stress (Supplementary S1). The content of BEA increased significantly in the treatment group, and the synthesis pathway of BEA was constructed on the basis of previous reports. Valine and phenylalanine were the precursors of BEA synthesis. During the synthesis of valine, pyruvate is the starting compound. The transcriptome showed that five genes related to the production of pyruvate increased significantly. Pyruvate is not only the precursor of valine, but also participates in the production of ATP [59]. Pyruvate’s highly upregulated transcriptional level will lead to the function of improving ATP generation [54,60]. Although there was no significant difference between the genes corresponding to acetolactate synthase (EC 2.2.1.6) and ketol-acid reductoisomerase (EC 1.1.1.86), the transcription level was higher than 92% of expressed genes. 2-Ketoisovaleric acid is a compound directly involved in the synthesis of BEA in the valine synthesis pathway (Supplementary Table S3). The synthesis gene of 2-ketoisovaleric acid was significantly upregulated, while the valine-producing gene was significantly inhibited. More 2-ketoisovaleric acid further synthesize 2-hydroxy-isovaleric acid to synthesize BEA. During the synthesis of phenylalanine, the transcription levels of all enzyme-corresponding genes from the starting compounds 4-erythrose phosphate and phosphoenolpyruvate to prephenic acid were significantly upregulated. The transcriptional level of the genes corresponding to the enzyme producing phenylpyruvate from prephenate (EC 4.2.1.51) and phenylalanine from phenylpyruvate (EC 2.6.1.57) were more than 80% of the expressed genes (Supplementary Table S3). The latter was reduced by 1.61 times. 2-Hydroxyisovaleric acid is a common 2-hydroxycarboxylic acid component in NRP such as BEA, Beauverolide, and Enniatins. The Kivr gene encodes a novel NADPH-dependent 2-ketoisovanoate reductase (KIVR), which converts 2-ketoisovanoate from valine decomposition or pyruvate metabolism to D-Hiv. In *B. bassiana*, Kivr knockdown completely inhibited the production of BEA and beauverolide, indicating that KIVR played a key role in the biosynthesis of BEA [61]. As the most important enzyme in the synthesis of BEA, it recognizes, activates, and connects phenylalanine and 2-hydroxyisovaleric acid to form a “dipeptide”, which is cyclized to form BEA after three cycles of reactions [33]. In the study, the gene was upregulated nearly 27 times in the H_2_O_2_ treatment compared with the control (Table 2), indicating that the enzyme was one of the rate-limiting enzymes for BEA synthesis. The result was consistent with the HPLC measurement of BEA. A previous report showed BbKIVR activity was suppressed under light and salt stress but not affected by oxidative stress in *B. bassiana* mycelia via the calmodulin (CaM) signaling pathway [62]. We found that oxidative stress promotes KIVR activity in *C. chanhua* mycelia. Further studies are required for addressing this question. This suggests that in the mass production of *C. chanhua*, weakening oxidative stress and suppressing of the activity of the enzymes by reducing oxygen content or adding edible antioxidants might be a prevention strategy.

## 5. Conclusions

On the basis of our metabolomic and transcriptomic analyses of *C. chanhua*, we find that NRPs, especially BEA, may be involved in *C. chanhua* response to oxidative stress. Given that BEA were upregulated significantly, it seems reasonable to speculate that BEA accumulation is in response to oxidative stress in *C. chanhua*. We suggest that *C. chanhua* responds to H_2_O_2_-induced oxidative stress by upregulating its own antioxidants, enhancing substance and energy metabolism, and regulating permeability of the plasma membrane. Enhancement of the glycolytic pathway and energy metabolism affect the pyruvate metabolism, and then affect the valine pathway and phenylalanine pathway, resulting in increased BEA synthesis. This knowledge about BEA will contribute to control BEA content and reduce potential food safety risks in *C. chanhua* utilization.

## Figures and Tables

**Table 1:** DEG enrichment pathways according to the KEGG database (*p* < 0.05).

Pathway ID	Pathway Name	Level 1	Level 2	DEGs	All Genes	Rich Ratio	*p*-Value
ko04011	MAPK signaling pathway—yeast	Environmental Information Processing	Signal transduction	296	2250	13.16%	0.0000151
ko00520	Amino sugar and nucleotide sugar metabolism	Metabolism	Carbohydrate metabolism	258	1934	13.34%	0.0000244
ko00920	Sulfur metabolism	Metabolism	Energy metabolism	20	78	25.64%	0.00014653
ko00600	Sphingolipid metabolism	Metabolism	Lipid metabolism	20	87	22.99%	0.000691245
ko00460	Cyanoamino acid metabolism	Metabolism	Metabolism of other amino acids	32	180	17.78%	0.002592972
ko00590	Arachidonic acid metabolism	Metabolism	Lipid metabolism	6	15	40%	0.003140305
ko01130	Biosynthesis of antibiotics	Metabolism	Global and overview maps	109	800	13.63%	0.0038334
ko00073	Cutin, suberine, and wax biosynthesis	Metabolism	Lipid metabolism	2	2	100%	0.01141278
ko00380	Tryptophan metabolism	Metabolism	Amino acid metabolism	28	169	16.57%	0.01216486
ko00620	Pyruvate metabolism	Metabolism	Carbohydrate metabolism	30	188	15.96%	0.0160634
ko00480	Glutathione metabolism	Metabolism	Metabolism of other amino acids	15	80	18.75%	0.02103369
ko01200	Carbon metabolism	Metabolism	Global and overview maps	51	360	14.17%	0.02131817
ko01230	Biosynthesis of amino acids	Metabolism	Global and overview maps	49	342	14.33%	0.01963021
ko00670	One carbon pool by folate	Metabolism	Metabolism of cofactors and vitamins	8	35	22.86%	0.0283667
ko00071	Fatty acid degradation	Metabolism	Lipid metabolism	22	140	15.71%	0.04119299

**Table 2:** Genes involved in BEA metabolism network.

Enzymes	EC Number	Gene ID	Transcriptional Level Treat/Control, Log_2_ Fold Change(Treat/Control)
Fructose-bisphosphate aldolase, class II	4.1.2.13	CL2518.Contig2	32.6/18.1, 0.90
2,3-Bisphosphoglycerate-independent phosphoglycerate mutase	5.4.2.12	CL863.Contig2	76.8/40.2, 0.96
Pyruvate kinase	2.7.1.40	Unigene4061	447.2/271.9, 0.76
D-Lactate dehydrogenase	1.1.2.4	CL800.Contig2	50.2/27.1, 0.93
Alanine transaminase	2.6.1.2	CL1908.Contig3	24.6/18.1, 0.49
L-Serine/L-threonine ammonia-lyase	4.3.1.17	Unigene1709	61.1/20.4, 1.58
Malate dehydrogenase	1.1.1.40	CL1818.Contig5	51.0/18.7, 1.47
Dihydroxy-acid dehydratase	4.2.1.9	Unigene5252	7.6/4.0, 0.99
		CL146.Contig5	2.3/1.1, 1.12
Branched-chain amino acid aminotransferase	2.6.1.42	CL4308.Contig1	40.4/54.5, −0.38
3-Deoxy-7-phosphoheptulonate synthase	2.5.1.54	CL2696.Contig4	93.2/45.8, 1.05
		CL2696.Contig3	169.6/45.7, 1.76
Pentafunctional AROM polypeptide	4.2.3.4	Unigene3610	38.2/20.0, 0.99
	4.2.1.10		
	1.1.1.25		
	2.7.1.71		
	2.5.1.19		
Chorismate synthase	4.2.3.5	Unigene4335	10.7/6.7, 0.72
Chorismate mutase	5.4.99.5	CL2040.Contig1	3.4/1.8, 0.90
Ketoisovalerate reductase	1.1.1.169	Unigene1653	42.9/1.6, 3.97
BEAS beauvericin nonribosomal cyclodepsipeptide synthetase	Fpbeas	Unigene6975	0.24/2.1, 2.47
	Fpbeas	Unigene3578	0.18/3.97, 2.54
	Fpbeas	Unigene2977	0.33/5.85, 3.33

## Data Availability

Not applicable.

## Supplementary Materials

The following supporting information can be downloaded at https://www.mdpi.com/article/10.3390/jof8050484/s1, S1: Free amino acids assay [63,64]. Table S1: Data filtering_ Filtered reads quality statistics. Table S2: Number of DEGs. Table S3: Expression level of genes.
