# Supplementary material for: Integration of Untargeted Metabolomics with Transcriptomics Provides Insights into Beauvericin Biosynthesis in Cordyceps chanhua under H2O2-Induced Oxidative Stress
Source: J Fungi (Basel). 2022 May 6;8(5):484. doi: 10.3390/jof8050484 (PMC9143143; doi:10.3390/jof8050484)
Supplement: Supplementary file 1 [file jof-08-00484-s001.zip › Supplementary S1. Free amino acids assay.pdf]

Free amino acids assay

Methods

200mg lyophilized mycelia powder was extracted with 4ml water [63] and filtered through 0.22μm PVDF membrane before HPLC–MS analysis. The mobile phases were distilled water (A, containing 0.1% formic acid) and acetonitrile (B, containing 0.1% formic acid); Elution conditions: 0-20min, 5% B; injection volume, 10 μL; flow rate, 0.2 ml/min; column temperature, 30℃, and the column was Agilent poroshell 120 EC-C18 column (2.7μm, 3.0 × 100mm). The eluent was monitored with a photodiode array detector, and the full-wavelength scan was performed from 200 to 600nm. The method of qualitative and quantitative analysis of 16 amino acid standard refers to Liu et al. [64]. The mass spectrometer parameter setting refers to Luo and He et al. (see reference [28, 29] in the manuscript) and were partially modified, the collection range is 50-1000 m/z. The acquisition range was 50-1000 m/z.

Results

14 free amino acids were detected while glycine and alanine not.

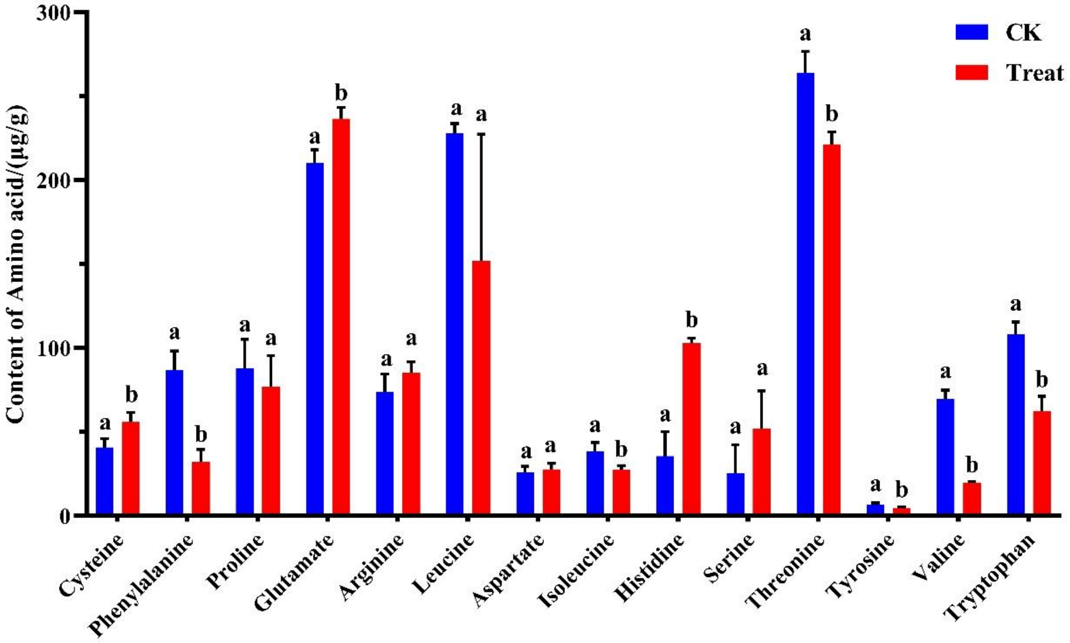

Figure S1. Contents of some free amino acids in mycelia of *C.chanhua*. The X-axis represents amino acids and the Y-axis represents the contents of amino acids. Blue, CK, without H_2O_2; Red, Treat, with H_2O_2. Different letters indicate that the difference is significant at the level of 0.05.
